# PTCD and choledochoscopy for recurrent choledocholithiasis after multiple abdominal surgeries: a case report

**DOI:** 10.3389/fmed.2024.1466184

**Published:** 2024-12-24

**Authors:** Liqiang Li, Zihan Zeng, Wenbo Li, Jun Lu, Liang Li, Jun Zhang

**Affiliations:** ^1^Department of General Surgery, The Second People’s Hospital of Hefei, Hefei Hospital Affiliated to Anhui Medical University, Hefei, China; ^2^Department of General Surgery, Hefei Second People’s Hospital Affiliated to Bengbu Medical University, Bengbu, China

**Keywords:** gallbladder stones, cholangitis, percutaneous transhepatic cholangiodrainage, case report, epigastric pain, history of multiple abdominal surgeries

## Abstract

**Background:**

Special attention should be given to intra-abdominal adhesions in patients with a history of open cholecystectomy for gallstones or abdominal surgery. Choosing the appropriate surgical approach to remove the stones is crucial.

**Patient summary:**

A 68-year-old male was admitted due to sudden onset of upper abdominal pain lasting more than 6 h. In 2018, he underwent open Billroth II surgery for gastric cancer at an external hospital, and in 2020, he underwent open cholecystectomy for gallstones. In August 2023, he received gamma knife treatment for recurrent gastric cancer brain metastasis at another hospital with good results. In December of the same year, the patient presented to our hospital due to recurrent common bile duct stones and cholangitis. Given his history of two abdominal surgeries, percutaneous transhepatic cholangiodrainage (PTCD) combined with choledochoscopic stone extraction was chosen, which was successful in completely removing the stones. A PTCD tube was left in place postoperatively.

**Conclusion:**

For patients with a history of two or more abdominal surgeries who experience recurrent common bile duct stones, PTCD has the advantages of a shorter operative time, less blood loss, earlier postoperative ventilation, earlier resumption of eating, minimal trauma and faster recovery.

## Introduction

The patient had a history of multiple laparotomies, with considerations of abdominal adhesions and poor physical condition. Therefore, percutaneous transhepatic cholangioscopy combined with lithotripsy, in which the bile duct is entered from top to bottom for stone extraction, was chosen. This approach offers advantages such as minimal trauma and rapid recovery.

Biliary tract stones are common clinical conditions characterized by a high incidence, high recurrence rate, and complexity (1, 2). Surgery is usually the main treatment modality. However, for complex biliary tract stones or patients with a history of multiple abdominal surgeries, the traditional surgical approach becomes more challenging, resulting in compromised treatment outcomes (3). For elderly patients with a history of multiple open abdominal surgeries who are reluctant to undergo another open surgery to remove common bile duct stones, ERCP is an option. However, when there are changes in biliary tract anatomy, such as bilioenterostomy, total or partial gastrectomy (such as Billroth II), or complicated biliary tract conditions accompanied by biliary tract stenosis, it can be difficult for the endoscope to enter the biliary duct through the nipple, rendering therapeutic relief with ERCP difficult (4). In this case, PTCD combined with choledochoscopy, which offers a significant advantage (5, 6). However, this approach increases the degree of technical difficulty for the surgeon. Additionally, in patients with severe abdominal adhesions, PTCD combined with choledochoscopy has become an even more suitable option (7). Therefore, when treating intrahepatic and extrahepatic biliary tract stones, it is necessary to choose a treatment method that is both effective and safe. With the development of minimally invasive surgery in recent years, there are diverse options for the treatment of complex biliary tract stones, such as those combined with biliary strictures or giant stones (8, 9). For complex common bile duct stones of this type, ERCP and EST procedures can be chosen (10). These procedures reduce surgical trauma, thereby minimizing surgical bleeding and shortening the operative time, which promotes postoperative recovery and shortens the hospital stay (11, 12). However, ERCP and EST procedures require an incision in the sphincter of Oddi, which may cause bile reflux in some patients, which is a lifelong issue that imposes significant mental and financial strain (13, 14). The main drawback of ERCP and EST lies in damage to the papillary sphincter, particularly in patients with larger stones; despite their minimally invasive appearance, these procedures cause substantial functional trauma (15–17). Therefore, PTCD combined with choledochoscopy for stone removal is the optimal choice for patients with a history of multiple abdominal surgeries (18). Under ultrasound guidance, PTCD combined with choledochoscopy offers a safe, simple, easy-to-master approach, allowing for one-time or staged tract establishment for stone extraction and lithotripsy (40, 19). This method has been safely and effectively applied in cases of complex bile duct stones, recurrent stones, diffuse hepatobiliary stones with severe cirrhosis, postoperative residual stones after bile duct exploration, bile duct obstruction due to strictures, and biliary purulent infections. Compared with traditional surgery, PTCD has significant advantages; specifically, stone extraction via hepatic puncture tunnels causes minimal trauma to the patient, provides greater stability during the procedure, and allows for the use of pneumatic lithotripsy and holmium laser lithotripsy for stones larger than 2 cm or impacted in the bile ducts (20–22). This ensures more thorough fragmentation and easier stone removal, shortening the operative time and increasing efficiency (23). For patients with residual stones or those requiring multiple sessions for complete stone removal, additional extraction can be performed 1 week later through the retained drainage tube, further improving the rate of complete clearance (24, 25). Percutaneous transhepatic cholangioscopy combined with lithotripsy is a minimally invasive surgery with advantages such as minimal trauma, quick recovery, and small incisions, showing great potential in clinical practice (26). This case involves a patient with a history of multiple abdominal surgeries and recurrent common bile duct stones, for whom percutaneous transhepatic cholangioscopy combined with lithotripsy was chosen as the optimal surgical approach.

## Case presentation

### Chief complaints

Sudden onset of upper abdominal pain/discomfort lasting for 6 h.

### History of present illness

The patient experienced sudden onset of upper abdominal pain/discomfort accompanied by nausea, without vomiting, chills, fever, palpitations, or chest tightness, 6 hours prior. The patient sought symptomatic treatment at a local hospital with no significant improvement and subsequently presented to our emergency department. Emergency abdominal computed tomography (CT) revealed common bile duct stones with biliary obstruction. The patient was admitted to our department with a provisional diagnosis of “(1) Common bile duct stones; (2) Common bile duct dilation.” Since the onset of symptoms, the patient has maintained good mental status, adequate sleep, normal appetite, regular bowel movements, normal urination, and no significant change in weight.

### History of past illness

In March 2018, the patient underwent open Billroth II subtotal gastrectomy at an outside hospital for gastric cancer. The surgery was successful, and the patient was discharged without any drains or tubes. In May 2020, the patient was hospitalized again for cholelithiasis with acute cholecystitis and underwent open cholecystectomy. According to the patient’s family, this surgery took a prolonged time, primarily because of severe intra-abdominal adhesions caused by previous gastric cancer surgeries, which made it difficult to locate and delineate the cystic duct and common bile duct.

In August 2023, the patient experienced metastasis of gastric cancer to the brain. At this point, open surgery was no longer feasible, so the patient underwent Gamma Knife radiosurgery at an outside hospital to treat the brain tumor. Gamma knife radiosurgery is a stereotactic radiotherapy technique with advantages such as minimal pain and a high localized radiation dose. It utilizes the principles of stereotactic geometry to selectively target intracranial lesions. The focused high-dose gamma rays produced by cobalt-60 induce double-strand breaks in the DNA of tumor cells, leading to tumor cell destruction, thereby increasing patient survival. Additionally, fractionated stereotactic radiotherapy with Gamma Knife for brain tumors has shown favorable efficacy, with high local control rates and reasonable long-term survival rates. It can also effectively improve patients’ emotional well-being.

### Personal and family history

There was no other relevant personal or family history.

### Physical examination

The patient’s body temperature was 36.5°C; heart rate, 52 bpm; respiratory rate, 21 breaths/min; fingertip oxygen saturation (SpO_2_), 96%; and body weight, 55 kg. The skin and sclerae were free from jaundice, and there was no enlargement of the supraclavicular lymph nodes. The abdomen was flat, and abdominal respiration was present. There were no abdominal wall varices or visible peristaltic waves. Bowel sounds were normal. No abdominal vascular murmurs were heard. Surgical scars were visible on the abdomen, with good healing. Upper abdominal tenderness was present, rebound tenderness was absent, and there was no guarding. The liver and spleen were not palpable below the costal margin. No shifting dullness was appreciated. Hepatic dullness was present without percussion tenderness. There was no renal percussion tenderness bilaterally.

### Laboratory examinations

Before the operation, the following biochemical tests were performed: total bilirubin, 23.74 μmol/L; alanine aminotransferase, 176 U/L; aspartate aminotransferase, 103 U/L; *γ*-glutamyltransferase, 568 U/L; alkaline phosphatase, 369.0 U/L; and creatine kinase, <20 U/L.

### Imaging examinations

**Figure 1:**
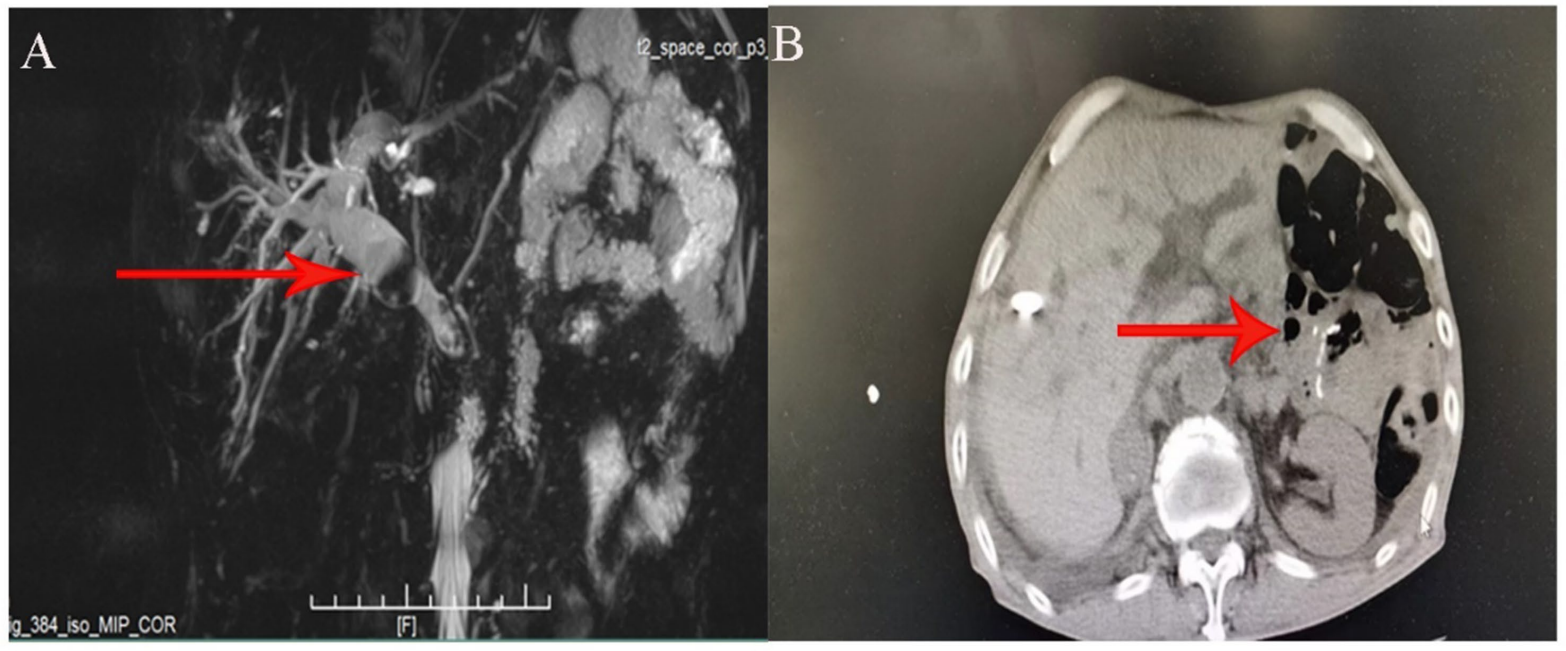
**(A)** Preoperative MRCP image showing the size and location of the common bile duct stone in the patient. **(B)** Pneumatosis and abdominal adhesion.

**Figure 2:**
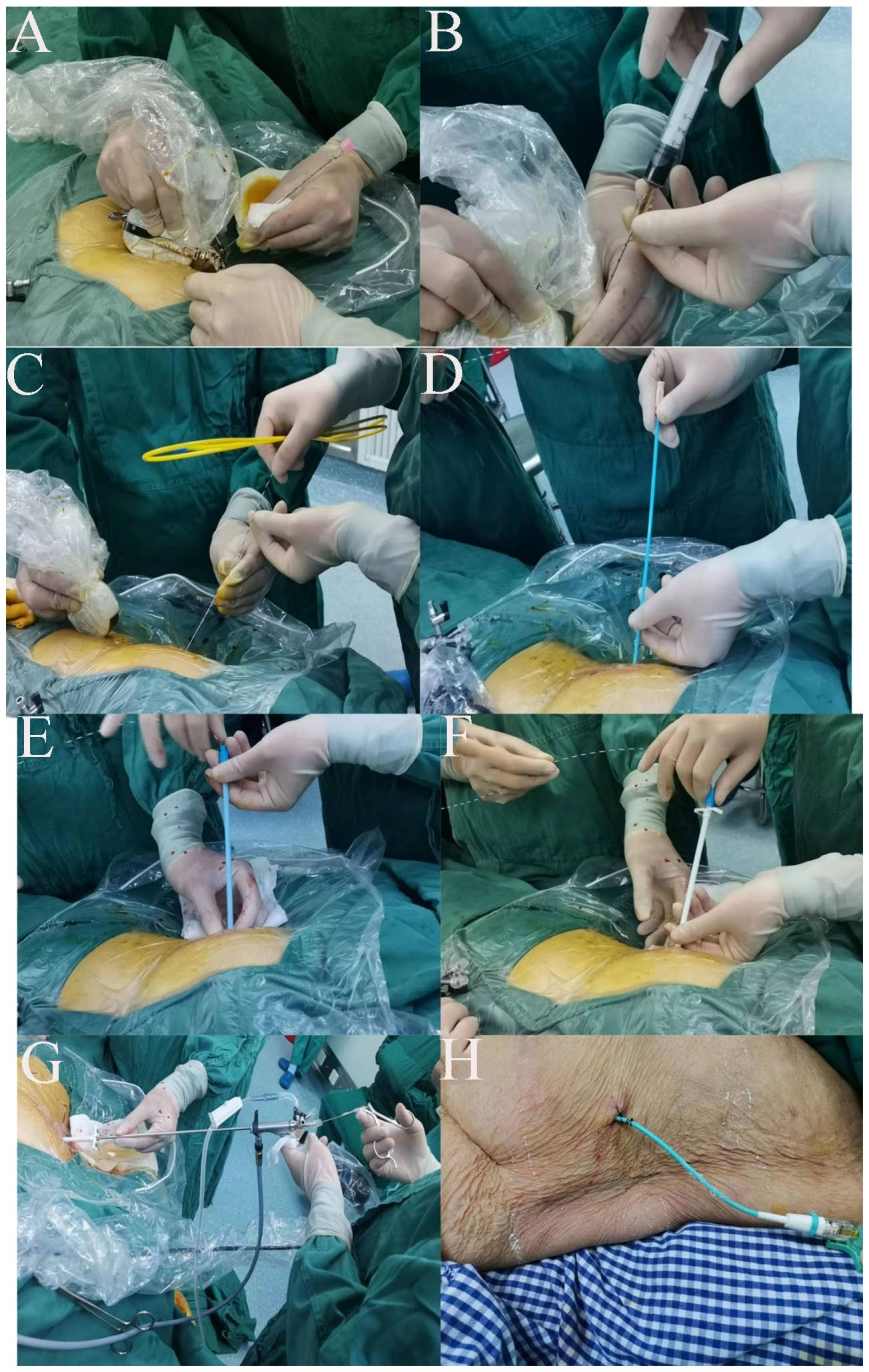
**(A)** Ultrasound guidance used to identify the dilated bile duct and determine the optimal skin entry point for needle insertion. **(B)** Insertion of a cannula needle (16G) into the dilated bile duct under ultrasound guidance. Aspiration of thick bile confirms correct needle placement within the bile duct. **(C)** Introduction of a J-tip guidewire through the cannula needle, followed by a controllable active core guidewire to secure access and maintain position within the bile duct. **(D)** Removal of the cannula needle and insertion of a dilator over the guidewire to gradually expand the puncture tract and create a pathway for the choledochoscope. **(E)** Progressive dilation of the tract using sequentially larger dilators to accommodate the rigid choledochoscope. **(F)** Final dilation achieved. The tract is now sufficiently wide for the introduction of the choledochoscope. **(G)** Introduction of the rigid choledochoscope through the dilated tract into the bile duct. **(H)** Postprocedure placement of an external drainage catheter (pigtail catheter) connected to the bile duct and secured to the skin.

1. A 68-year-old male patient presented with sudden upper abdominal pain lasting for more than 6 h. Magnetic resonance cholangiopancreatography (MRCP) upon admission revealed a stone at the lower end of the common bile duct measuring approximately 1.5*1.5 cm (Figure 1A). The patient had undergone open radical surgery for gastric cancer (subtotal gastrectomy) in 2018. In 2020, he underwent open cholecystectomy due to gallbladder stones. Prior to undergoing PTCD combined with choledochoscopy stone extraction, the patient had undergone gamma knife treatment for brain metastasis of gastric cancer at an external hospital (Figure 1B).
2. PTCD combined with a choledochoscopy stone extraction procedure: After administering general anesthesia, routine disinfection and draping were performed. Under ultrasound guidance, bile duct dilation was confirmed, and a skin incision of 3–4 mm was made. Using ultrasound guidance, a cannula needle (16G) was inserted into the identified bile duct (Figure 2A). The needle core was removed, and a syringe was attached for slow aspiration. If thick bile was drawn out, the cannula was positioned inside the bile duct (Figure 2B). A J-tip guidewire and a controllable active core guidewire were subsequently introduced through the cannula to ensure proper positioning within the bile duct. The cannula was then replaced with a dilator to expand the puncture path (Figures 2B–F).

**Figure 3:**
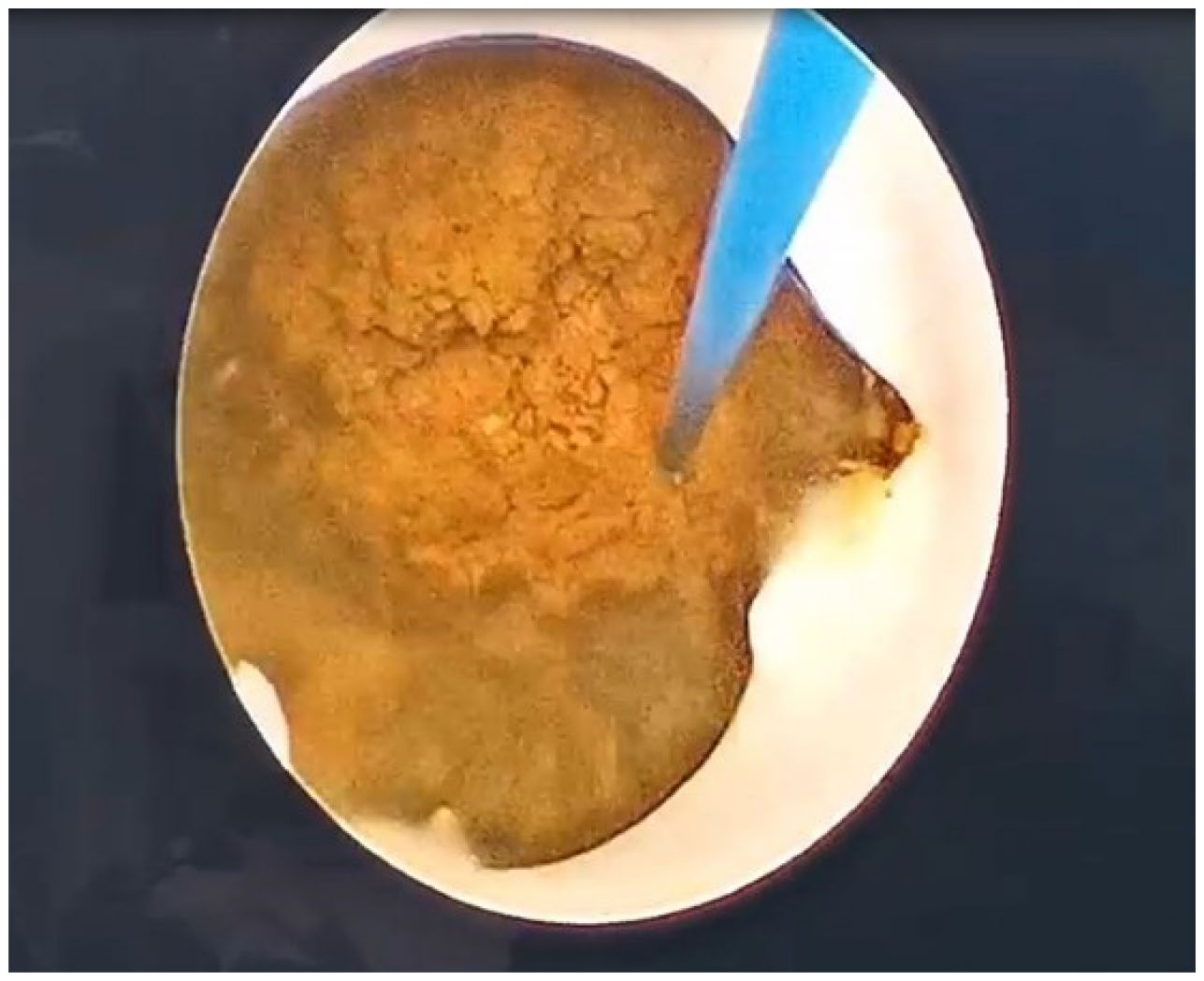
Stone under the choledochoscope.

At this point, a rigid choledochoscope (Insert a rigid choledochoscope through the sheath to explore for common bile duct stones. For stones smaller than the diameter of the sheath, use stone forceps to grasp them. Larger stones can be fragmented using a holmium laser and then flushed out with water through the sheath.) was introduced through the dilated tract (Figure 2G), and a holmium laser was connected to identify common bile duct stones. For stones smaller than the diameter of the sheath, stone forceps are used to grasp them. Larger stones can be fragmented via a holmium laser and then flushed out with water through the sheath. The holmium laser energy was dynamically adjusted to fragment visible stones continuously within the bile duct (Figure 3). Continuous flushing with irrigation solution was performed during stone fragmentation. After successful completion of the procedure, an external drainage catheter was connected to the patient (Figure 2H) and secured in place.

### Final diagnosis

Common bile duct stones, common bile duct dilation, personal history of gastric malignancy, and postcholecystectomy status.

### Treatment

The patient underwent PTCD combined with choledochoscopic stone extraction, which resulted in minimal trauma and rapid recovery.

### Outcome and follow-up

The patient was discharged after 10 days of postoperative hospitalization, with approximately 200–300 mL of bile drainage per day and no observed abnormal color or characteristics. Liver function and other tests were performed on the third day after surgery, and the following results were obtained: total bilirubin, 14.7 μmol/L; alanine aminotransferase, 20 U/L; aspartate aminotransferase, 7.5 U/L; *γ*-glutamyltransferase, 381 U/L; alkaline phosphatase, 222.3 U/L; and creatine kinase, 28 U/L. The patient’s liver function and color and characteristics of bile drainage from the drain were normal, leading to discharge on the sixth day. The patient will retain the PTCD tube (pigtail catheter) for an additional 2 months after discharge and will return to the hepatobiliary surgery department for tube removal at the end of this period. During the tube retention period, the patient took care to protect the PTCD tube, and no complications, such as infection, stone recurrence, or dislodgement, were observed. The PTCD tube was removed upon returning to the hospital after 2 months, and liver function was reassessed. Two months later, the patient returned to the hospital for removal of the T-tube and reexamination of the following biochemical results: total bilirubin, 8.7 μmol/L; alanine aminotransferase, 19 U/L; aspartate aminotransferase, 34 U/L; *γ*-glutamyltransferase, 54 U/L; alkaline phosphatase, 116 U/L; and creatine kinase, 48 U/L.

## Discussion

PTCD combined with choledochoscopy stone removal surgery has the same purpose as LCBDE, which is to establish a pathway for stone removal (27, 28). However, PTCD combined with choledochoscopy aims for a simpler puncture operation and implements a “top-down” stone removal approach, effectively avoiding the surgical risks associated with LCBDE and addressing the difficulty of accessing narrowed intrahepatic bile duct openings (28–30). PTCD combined with choledochoscopy stone removal is intended for cases of recurrent bile duct stones that are difficult to manage with conventional surgery. This includes a history of multiple bile duct explorations and stone removal procedures, extensive adhesions at the hepatic hilum, unclear ductal structures, or patients who have undergone multiple surgeries without a cure and refuse further surgical treatment (31, 32). It also applies to patients with a history of complex upper abdominal surgeries, such as total gastrectomy, with extensive intra-abdominal adhesions. PTCD combined with choledochoscopy allows for a clever top-down entry into the bile duct, avoiding the risks and injuries associated with LCBDE (20). However, PTCD combined with choledochoscopy also has limitations, such as cases involving nonrecurrent bile duct stones or bile duct stones combined with hepatic lobe atrophy, for which conventional hepatobiliary surgery methods such as LCBDE remain the preferred choice (23, 25).

Compared with traditional open common bile duct exploration for stone removal, LCBDE offers advantages of less trauma and faster recovery (33). PTCD combined with choledochoscopy can also be used for common bile duct stone extraction. LCBDE has strict requirements for the common bile duct diameter, specifically >8 mm, as a narrower duct may lead to tearing upon choledochoscope insertion, potentially causing severe biliary bleeding (34). Additionally, studies have reported a low clearance rate for intrahepatic bile duct stones with LCBDE, with residual stone rates reaching up to 87.3% (35, 36).

Research has revealed no significant difference in stone clearance efficacy or complication rates between PTCD combined with choledochoscopy and LCBDE (37). However, PTCD with choledochoscopy has significantly shorter operative times. Han suggested that LCBDE may cause damage to adhesion bands, resulting in greater intraoperative blood loss (37). PTCD combined with choledochoscopy, however, accesses stones directly through percutaneous liver puncture, reducing organ trauma and thus leading to less bleeding compared with common bile duct exploration.

These limitations restrict the application of LCBDE in treating intrahepatic bile duct stones. As PTCD with choledochoscopy technology advances, more health care institutions are adopting PTCD as a primary treatment method. This approach is particularly beneficial for patients with extensive intra-abdominal adhesions due to multiple prior open abdominal surgeries, as either repeat laparotomy or LCBDE increases the difficulty and risk of surgery, increasing financial and psychological stress for the patient.

Compared with traditional surgery, PTCD combined with choledochoscopy stone removal offers significant advantages. Stone retrieval through a hepatic puncture tunnel not only minimizes patient injury but also provides easy intraoperative manipulation. Additionally, for patients with bile duct stones larger than 2 cm or stones impacted in the duct, holmium laser lithotripsy can be employed during surgery, facilitating easier stone extraction, thereby reducing operative time and enhancing surgical efficiency (38, 39). In this case, holmium laser lithotripsy was utilized for stone fragmentation, making stone retrieval easier. Particularly for patients with stones that cannot be completely removed in one procedure or with residual stones, stone retrieval can be performed again through the indwelling drainage tube 1 week later, thus increasing the clearance rate.

From this case, we can observe that PTCD combined with choledochoscopy stone removal enables precise localization, allowing for the selection of the optimal bile duct entry route. In this case, we utilized an electronic choledochoscope, which offers advantages such as flexible operation, clear visualization, and multiangle maneuverability, facilitating exploration of the majority of intrahepatic and extrahepatic bile ducts via suitable single or dual channels. In contrast, post-LCBDE stone retrieval via the sinus tract requires upward exploration of the intrahepatic bile ducts through the common bile duct to locate the stones, thereby reducing stone retrieval efficiency. PTCD combined with choledochoscopy establishes a channel between the bile duct and the outside of the body via a sheath, which facilitates stone extraction and optimizes the surgical procedure. The use of a choledochoscope also helps improve the stone clearance rate, ensuring effective treatment outcomes. However, PTCD combined with choledochoscopy stone removal surgery also has limitations (41–43), as follows. (1) There are high technical demands on the operator, as selecting the bile duct puncture point, ultrasound-guided puncture techniques, and sinus tract dilation techniques all require advanced skills. (2) There is a continued risk of bleeding: intraoperative bleeding often occurs during bile duct puncture, sinus tract dilation, and stone retrieval. Forceful dragging of large stones during basket retrieval can lead to sheath slippage, bile duct wall damage, violent dilation of narrowed bile ducts, and subsequent bleeding. (3) There is excessive intraoperative irrigation: continuous infusion of saline solution is required during bile duct stone retrieval, and due to the lengthy surgical duration, a large volume of saline solution may accumulate in the patient’s intestines, leading to postoperative abdominal distension, nausea, and potentially sepsis.

Stone extraction via PTCD combined with choledochoscopy not only requires a high level of technical expertise but also carries risks of complications. For example, during the puncture procedure, complications such as pleural effusion, empyema, or subphrenic abscess may arise, primarily due to PTCD puncturing the upper bile ducts near the hepatic-diaphragmatic surface. If these complications occur, immediate antibiotic therapy or pleural puncture drainage is needed; in severe cases, thoracotomy may be necessary.

Biliary bleeding is another potential complication, typically resulting from holmium laser lithotripsy within the bile duct. If biliary bleeding occurs, the procedure should be stopped immediately. Minor bleeding may be managed conservatively, but persistent or significant bleeding requires symptomatic treatment, such as laparoscopic suturing of the common bile duct to control the hemorrhage.

In cases of PTCD tube dislodgement, if bile peritonitis develops, emergency surgery is required to prevent further infection and reduce the risk of mortality.

## Conclusion

From this case, we can conclude that PTCD combined with choledochoscopy for stone extraction offers several advantages, including shorter hospital stays, high stone removal efficiency, shorter treatment cycles, and minimal invasiveness. This approach is especially beneficial for elderly patients with a history of multiple abdominal surgeries, where severe adhesions make laparoscopic or other minimally invasive techniques unfeasible, as well as for those who prefer to avoid open surgery. Moreover, the combination of PTCD and direct percutaneous transhepatic cholangioscopy for stone extraction minimizes organ damage, resulting in reduced bleeding in patients.

## Data Availability

The original contributions presented in the study are included in the article/supplementary material, further inquiries can be directed to the corresponding author.
